# The Protective Effect of *Auricularia cornea* var. Li. Polysaccharide on Alcoholic Liver Disease and Its Effect on Intestinal Microbiota

**DOI:** 10.3390/molecules28248003

**Published:** 2023-12-08

**Authors:** Tianci Wang, Zikun Jia, Canghai An, Ping Ren, Yiting Yang, Wanting Wang, Ling Su

**Affiliations:** 1 Engineering Research Center of Chinese Ministry of Education for Edible and Medicinal Fungi, Jilin Agricultural University, Changchun 130118, China; wangtianci0617@163.com (T.W.); jiazikun0728@163.com (Z.J.); 2 College of Plant Protection, Jilin Agricultural University, Changchun 130118, China; a19958500015@163.com; 3 Engineering Research Center of Bioreactor and Pharmaceutical Development, Ministry of Education, Jilin Agricultural University, Changchun 130118, China

**Keywords:** *Auricularia cornea* var. Li., polysaccharide, ALD, intestinal microbiota

## Abstract

This study’s objective was to examine the protective effect and mechanism of a novel polysaccharide (AYP) from *Auricularia cornea* var. Li. on alcoholic liver disease in mice. AYP was extracted from the fruiting bodies of *Auricularia cornea* var. Li. by enzymatic extraction and purified by DEAE-52 and Sephacryl S-400. Structural features were determined using high-performance liquid chromatography, ion exchange chromatography and Fourier-transform infrared analysis. Additionally, alcoholic liver disease (ALD) mice were established to explore the hepatoprotective activity of AYP (50, 100 and 200 mg/kg/d). Here, our results showed that AYP presented high purity with a molecular weight of 4.64 × 10^5^ Da. AYP was composed of galacturonic acid, galactose, glucose, arabinose, mannose, xylose, rhamnose, ribos, glucuronic acid and fucose (molar ratio: 39.5:32.9:23.6:18.3:6.5:5.8:5.8:3.3:2:1.1). Notably, AYP remarkably reduced liver function impairment (alanine aminotransferase (ALT), aspartate aminotransferase (AST), triglyceride (TG), total cholesterol (TC)), nitric oxide (NO) and malondialdehyde (MDA) of the liver and enhanced the activity of antioxidant enzymes (superoxide dismutase (SOD), glutathione peroxidase (GSH-Px) and glutathione (gGSH)) in mice with ALD. Meanwhile, the serum level of tumor necrosis factor-α (TNF-α), interleukin-6 (IL-6) and interleukin-1β (IL-1β) were reduced in ALD mice treated by AYP. Furthermore, the AYPH group was the most effective and was therefore chosen to further investigate its effect on the intestinal microbiota (bacteria and fungi) of ALD mice. Based on 16s rRNA and ITS-1 sequencing data, AYP influenced the homeostasis of intestinal microbiota to mitigate the damage of ALD mice, possibly by raising the abundance of favorable microbiota (*Muribaculaceae*, *Lachnospiraceae* and *Kazachstania*) and diminishing the abundance of detrimental microbiota (*Lactobacillus*, *Mortierella* and *Candida*). This discovery opens new possibilities for investigating physiological activity in *A. cornea* var. Li. and provides theoretical references for natural liver-protecting medication research.

## 1. Introduction

Alcoholic liver disease (ALD) is liver damage caused by long-term excessive alcohol consumption. It initially manifests as significant hepatocyte steatosis, which can progress to steatohepatitis, liver fibrosis and cirrhosis [1,2]. From 2017 to 2022, the related mortality rate of ALD has increased every year and has caused huge economic losses [3]. At present, the study of ALD treatment has become a global research hotspot. Although ALD has a profound harmful impact, due to its complex pathogenesis such as oxidative stress [4,5] and cytokine-mediated inflammation [5], etc., little progress has been made in the treatment of ALD. The emergence of the intestinal–liver axis provides new ideas on the pathogenesis of ALD, and the control of microbiota is critical to maintaining homeostasis of the intestinal–liver axis [6]. However, when the host overtakes ethanol, the intestinal microbiota is disturbed. The intestinal bacteria community is highly sensitive to ethanol; for example, the abundance of *Proteobacteria* was increased, while the abundances of *Firmicutes* and *Bacteroides* were decreased in the intestinal bacteria of ALD animals and patients with ALD without cirrhosis [7]. In addition to the intestinal bacteria, the intestinal fungi are also altered in ALD, and systemic exposure to mycobiota correlates with the severity of liver damage. The role and mechanism of commensal fungi in the development of ALD were initially investigated. One is dependent on the C-type lectin domain family 7 member A (CLEC7A) pathway of Kupffer cells in livers, and another is mainly associated with fungi metabolite [8]. Consequently, modulation of the intestinal microbiota is a pertinent treatment target for ALD [9].

The most common medications for ALD are classified into three categories: supplemental raw materials for liver cell metabolism, opioid receptor antagonists and agents that manage and improve alcohol metabolism, which can cause severe adverse effects [10]. Therefore, it is necessary to explore safe and effective natural products with hepatoprotective activity. Numerous studies have shown that targeting intestinal microbiota may be one of the major underlying mechanisms of natural polysaccharides on ALD [11,12]. Polysaccharides from *Crassostrea gigas* attenuated ALD in mice by modulating intestinal bacteria [13]. Furthermore, polysaccharides from *Wolfporia cocos* ameliorated ALD in mice by modulating the composition of intestinal bacteria and reducing the abundance of harmful fungi [6]. Several natural polysaccharides from *Sporidiobolus pararoseus* [14] and *Morchella esculenta* [15] have the same mechanism for ALD treatment.

*Auricularia cornea* var. Li. (*A. cornea* var. Li.), a white-body edible fungi that belongs to the basidiomycetes and has both nutritional and medicinal value, is a variant of *A. cornea* [16]. As the main bioactive compounds, *A. cornea* var. Li. polysaccharide has antioxidant [17], anti-diabetic [18], immunomodulatory [19] and hepatoprotective effects [4]. Previous studies have reported that the crude polysaccharide of *A. cornea* var. Li. had the strong protective effect of against alcoholic liver injury [4]. However, it is hard to clarify the effect and mechanism of *A. cornea* var. Li. polysaccharide, and its role in intestinal microbiota on mitigating ALD is not clear.

In this research, a novel homogeneous polysaccharide enzymatically extracted from *A. cornea* var. Li. was obtained after purification by DEAE-52 and Sephacryl S-400. In the meantime, the ameliorative effect of AYP on ALD was investigated by designing a mouse model of acute ALD. Specifically, the roles of intestinal microbiota (bacteria and fungi) in the protective effect of AYP on ALD mice are discussed. Our work provides a new direction in the prevention of alcohol-related diseases by natural polysaccharides. 

## 2. Results and Discussion

### 2.1. Identification and Charactrization of AYP

**Figure 1:**
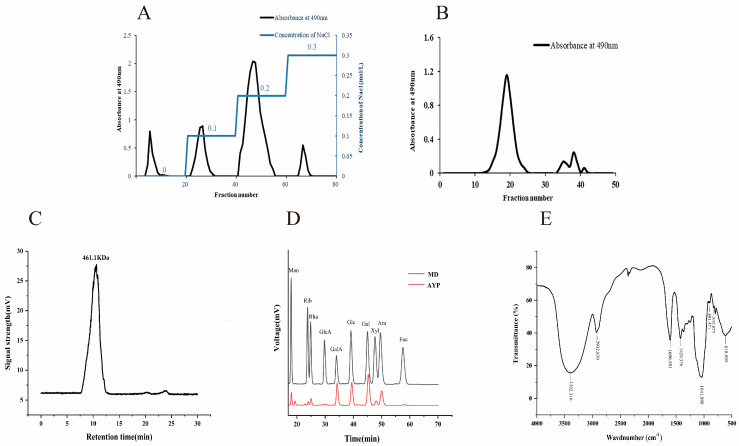
Characteristic analysis of the polysaccharide isolated from *Auricularia cornea* var. Li. DEAE-52 column elution curve of AYP (**A**), Sephacryl S-400 column elution curve of AYP (**B**), molecular weight peak of AYP (**C**), monosaccharide composition analysis of AYP (**D**), FT-IR spectra of AYP (**E**).

AYP was purified by DEAE-52 and Sephacryl S-400, as depicted in Figure 1A,B, respectively. Four fractions eluted with 0, 0.1, 0.2 and 0.3 mol/L NaCl solutions were collected, and the polysaccharide produced from 0.2 mol/L NaCl was re-eluted by Sephacryl S-400 and utilized in following research. The total polysaccharide content of AYP was 86.51 ± 0.15%, the reducing sugar content was 0.657 ± 0.48% and the protein content was 3.164 ± 0.05%, from which can be reasonably inferred that the purification in the present work was adequate for the follow-up research.

As shown in Figure 1C, a single symmetrical peak was observed in HPGPC profiles, verifying that AYP was a uniform polysaccharide with a molecular weight of 4.64 × 10^5^ Da, which was lower than that of the three fractional polysaccharides ultrasound-assisted extracted from *A. cornea* var. Li (ACPN-1a: 2.18 × 10^6^ Da, ACPA-2a: 8.5×10^5^ Da, ACPA-1a: 5 × 10^5^–2 × 10^6^ Da) [20]. Not coincidentally, the molecular weight of polysaccharides extracted from *Ginkgo biloba* seed by the enzymatic extraction method was lower than that of the ultrasound-assisted extraction, which was related to the fact that enzymatic extraction facilitated polysaccharide entry into the extraction solvent through enzymatic digestion [21]. 

AYP was a heteropolysaccharide, which was composed of galacturonic acid, galactose, glucose, arabinose, mannose, xylose, rhamnose, ribos, glucuronic acid and fucose in a molar ratio of 39.5:32.9:23.6:18.3:6.5:5.8:5.8:3.3:2:1.1 according to the HPLC result (Figure 1D). In contrast, EAPS, also obtained by the enzymatic extraction of *A. cornea* var. Li., consisted of Fuc, Rib, Xyl, Man, Gal and Glu in a molar ratio of 8.8:1.0:26.4:8.2:10.0:58.1 [4]. The proportion of GalA in the monosaccharide composition of AYP was much higher than that in EAPS, maybe due to the high temperature (85 °C) in our study, which is in line with a previous report that the highest extraction rate of GalA in grapevine pectin was achieved at 90 °C [22]. Therefore, AYP could be a novel polysaccharide different from EAPS from the perspective of monosaccharide composition.

The FT-IR of AYP is depicted in Figure 1E. -OH exhibited a stretching vibration at a wavelength of 3400 cm^−1^ [23], and a relatively robust absorption peak at approximately 1650 cm^−1^ also indicated the characteristic polysaccharide absorption [24]. At 1420 cm^−1^, there was another stretching vibration caused by C-H, and the characteristic absorption peak between 1020 cm^−1^ and 1230 cm^−1^ was the stretching vibration caused by C-O-C and C-OH [25]. The faint bands at 891 cm^−1^ and 832 cm^−1^ indicated the presence of β-linked and α-linked sugar residues, respectively [26]. It was hypothesized that AYP is a pyranose polysaccharide with both α and β conformations. In conclusion, a new homogeneous polysaccharide (AYP) obtained from *A. cornea* var. Li. had a lower molecular weight than that of *A. cornea* var. Li. polysaccharides extracted by the ultrasound method. Moreover, lower-molecular-weight polysaccharides have stronger biological activities. Thus, it is reasonable to speculate that AYP may have a stronger hepatoprotective effect.

### 2.2. The Effect of AYP on the Liver Damage of Mice 

**Figure 2:**
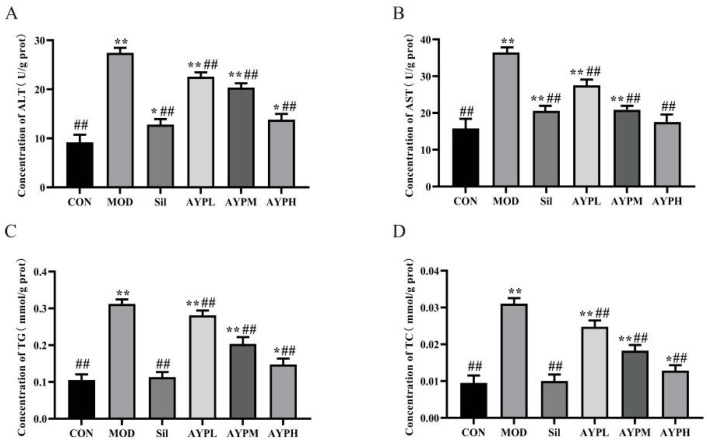
The effect of AYP on the liver function indexes of mice. ALT (**A**), AST (**B**), TG (**C**) and TC (**D**). * *p* < 0.05, ** *p* < 0.01 vs. CON; ## *p* < 0.01 vs. MOD. Duncan’s test was performed to determine the differences.

**Figure 3:**
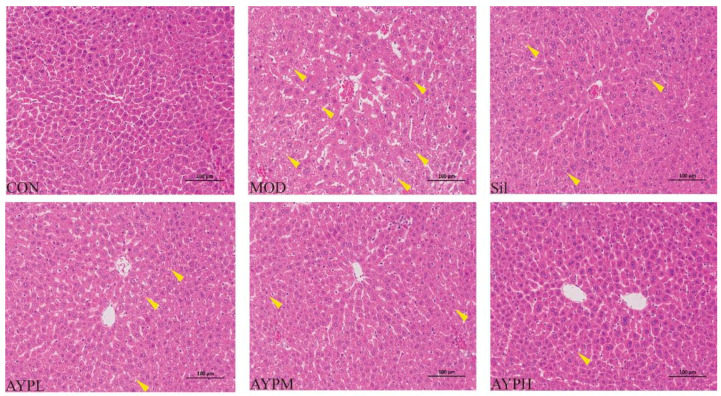
The histological sections of mice liver (200×). CON group, MOD group, Sil group, AYPL group, AYPM group and AYPH group, respectively.

Liver function (AST and ALT) and lipid metabolism (TG and TC) in serum are extremely correlated with the severity of ALD and serve as the main indicators for determining whether the ALD model has been successfully validated [27,28,29]. AST, ALT, TG and TC in the MOD group were significantly higher than the CON group (*p* < 0.01), suggesting that alcohol caused injury to the liver. ALT, AST, TC and TG levels in the livers of the AYP and Sil groups were lower than those of the MOD group (*p* < 0.01) (Figure 2). A previous study reported that *Echinacea* polysaccharide (EPP80), which was extracted from *Echinacea purpurea*, also prevented ALD in mice, but only ALT in the EPP80-L (100 mg/kg/d) group was significantly dissimilar to the MOD group (*p* < 0.05), and the AST, TG and TC indexes had no significant differences [30]. Therefore, it also indirectly indicates that the ability to alleviate the ALD of AYP may be more potent than that of EPP80. Polysaccharides with the highest percentage of GalA has proved to exist the highest antioxidant activity [31]. Hence, we speculated that AYP had a more effective protective activity than EPP80, which may be due to the different molar percentages of GalA in the polysaccharide composition, with 3.4% GalA in EPP80 [30] and 28.4% in AYP. To further confirm the hepatoprotective activity of AYP, HE staining of the liver tissue was conducted. Normal liver exhibited hepatic cell cords in orderly arrangements, distinct nuclei and well-defined cell borders in the CON group (Figure 3). Compared with the CON group, the MOD group showed severe liver damage as characterized by the loss of cellular boundaries, an indistinct hepatoplate, cellular degeneration and evident aggregates of lipid-droplet-like vacuoles, which were seen in most of the hepatocytes (yellow arrow). AYP and Silymarin led to a significant improvement in these histopathologies, as evidenced by the diminution of vacuolated cells and the increased integrity of cellular boundaries, as well as decreases in the cell volume and the number of lipid droplet-like vacuolar aggregates visible in the cytoplasm of hepatocytes (yellow arrow). Particularly in the AYPH group (200 mg/kg/d), the hepatic architectures were similar to those of the CON group. The above data further confirm that AYP had a noticeable improvement effect on the liver cells of mice with ALD.

### 2.3. The Effect of AYP on the Oxidation Indicators in Mice Liver 

**Figure 4:**
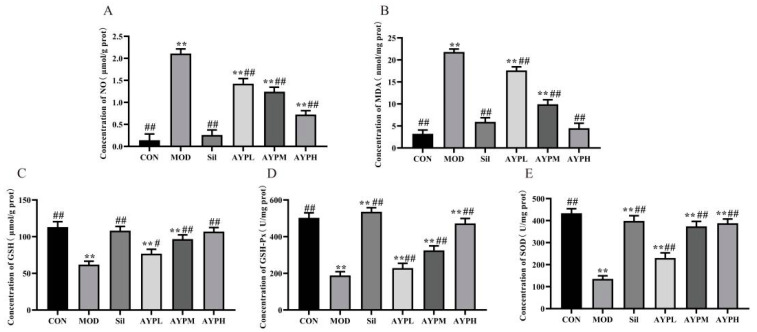
The effect of AYP on the contents of NO (**A**), MDA (**B**) and the activities of GSH (**C**), GSH-Px (**D**) and SOD (**E**) in the liver of mice. ** *p* < 0.01 vs. CON; # *p* < 0.05, ## *p* < 0.01 vs. MOD. Duncan’s test was performed to determine the differences.

NO, MDA, SOD, GSH-Px and GSH are significant indicators of the antioxidant status of an organism [32,33]. In this study, alcohol significantly decreased the activities of SOD (*p* < 0.01), GSH (*p* < 0.01) and GSH-Px (*p* < 0.01) and significantly increased the contents of MDA and NO (*p* < 0.01). In order to prevent the pathological development of ALD, increasing the activities of GSH, GSH-Px and SOD in the liver was beneficial in lowering the production of reactive oxygen species caused by excessive alcohol consumption [34]. Compared to the MOD group, the levels of NO and MDA were decreased dose-dependently in AYP and Sil groups, while AYP and Sil increased the activities of SOD, GSH and GSH-Px (Figure 4). Polysaccharide from *Lepidium meyenii* (MP-1) also had a defending effect on alcohol-induced oxidative liver damage in mice, but SOD and GSH-Px in the MP-1L (200 mg/kg/d) group had no significant differences from the MOD group [35]. Whereas GSH-Px in the AYPL (50 mg/kg/d) group had significant differences from the MOD group (*p* < 0.05). The molecular weight may be the key, which is generally inversely proportional to the activity [36]. Similarly, *Sophorae tonkinensis* Radix yielded two polysaccharides, STRP1 and STRP2, with average molecular weights of 1.30 × 10^4^ and 1.98 × 10^5^ Da, respectively. The potential liver-protective effects of STRP1 were more potent than those of STRP2 against acetaminophen-induced liver damage in mice [37]. Hence, we speculated that AYP had more effective protective activity than MP-1, which may be due to the different molecular weights, with 1.06 × 10^6^ Da molecular weight in MP-1 [35] and 4.64 × 10^5^ Da in AYP.

### 2.4. The Effect of AYP on the Secretion of Serum Cytokines in Mice 

**Figure 5:**
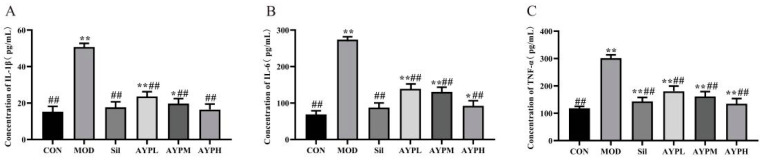
Effect of AYP on IL-1β (**A**), IL-6 (**B**) and TNF-α (**C**) levels induced by alcohol. * *p* < 0.05, ** *p* < 0.01 vs. CON; ## *p* < 0.01 vs. MOD. Duncan’s test was performed to determine the differences.

IL-6, TNF-α and IL-1β, which are crucial in the pathophysiology of ALD, can be overproduced when Kupffer cells are activated by alcohol-induced endotoxin [37]. Among these, IL-6 can lead to inflammation, fat buildup and liver tissue fibrosis [38]. Fibroblast degradation and deposition can be produced by IL-1β [39], and TNF-α may trigger inflammation by activating mononuclear macrophages [40]. The levels of three significant serum cytokines (IL-6, TNF-α and IL-1β) were examined in the plasma to assess whether AYP had the potential anti-inflammatory effects of IL-1β, TNF-α and IL-6, which were consistent with the fact that the polysaccharide from *Rosa rugosa* significantly reduced the production of inflammatory cytokines (Figure 5) induced by alcohol [5]. Therefore, AYP may also have the capability to protect against ALD by inhibiting pro-inflammatory mediators.

### 2.5. The Effect of AYP on the Intestinal Microbiota of Mice 

Since the concept of the enterohepatic axis was introduced, more and more studies have confirmed the role played by the intestinal microbiota in ALD [41]. Therefore, this study investigated the role of intestinal microbiota in ALD in terms of both intestinal bacteria and intestinal fungi.

#### 2.5.1. The Effect of AYP on the Intestinal Bacteria of Mice

The liver is the most exposed to potentially bacterial products or metabolites [42]. The pathogenesis of ALD is associated with intestinal bacterial disorders [43]. For instance, patients with alcoholic cirrhosis had bacterial hyperplasia and bacterial abnormalities in the small intestine [44]. This may be due to the fact that alcohol promotes the growth of hazardous bacteria and upsets the original equilibrium of intestinal bacteria [45].

According to Figure S1A, the dilution curved for all samples tended toward asymptotes, indicating that the sequencing data covered the vast majority of bacterial diversity. The OTU Venn diagram was created under the condition of 97% similarity to analyze the intestinal bacteria composition in mice. In total, 453 OTUs were found in the four categories and could be performed normally for the next analysis (Figure S1B). Furthermore, the rank abundance curve revealed that the sample species were abundant and consistent (Figure S1C).

Alpha diversity refers to the analysis of biodiversity within a particular area or ecosystem, which consists predominantly of the calculation of diversity indices such as the Chao1 richness index, Shannon diversity index and Simpson index [46]. The ACE and Chao1 indices are utilized to represent the species in the sample [47]. The Shannon index and the Simpson index can both be used to measure the diversity of bacteria. 

There was a highly significant difference (*p* < 0.01) in the Simpson and Shannon indices between the MOD and CON groups (Table 1). There was no statistical change in the ACE and Chao1 indices, but indices in the MOD group rose visually. These results support that alcohol consumption increased the number and decreased the diversity of intestinal bacteria [48]. There were highly significant differences (*p* < 0.01) in the ACE, Chao1 and Simpson indices among the Sil group, the AYP group and the MOD group, and there were significant differences (*p* < 0.05) in the Shannon indices between the AYP group and the MOD group, indicating that AYP affected the abundance and diversity of intestinal bacteria in ALD mice. The similarity of different samples in species diversity can be compared through β diversity (PCA and PLS-DA) analysis [49]. Following alcohol induction, PLS-DA and PCA demonstrated that the MOD group and CON group were well differentiated, indicating that alcohol modified the structure of intestinal bacteria. The AYP group had a significantly distinct composition of intestinal bacteria with the MOD group but was comparable to the CON and Sil groups (Figure S1D,E). Similarly, Dendrobium leaf extract enhanced α diversity and β diversity results in intestinal bacteria in ALD rats [50]. Therefore, α and β diversity suggest that AYP could prevent the changes of intestinal bacteria caused by ALD.

**Figure 6:**
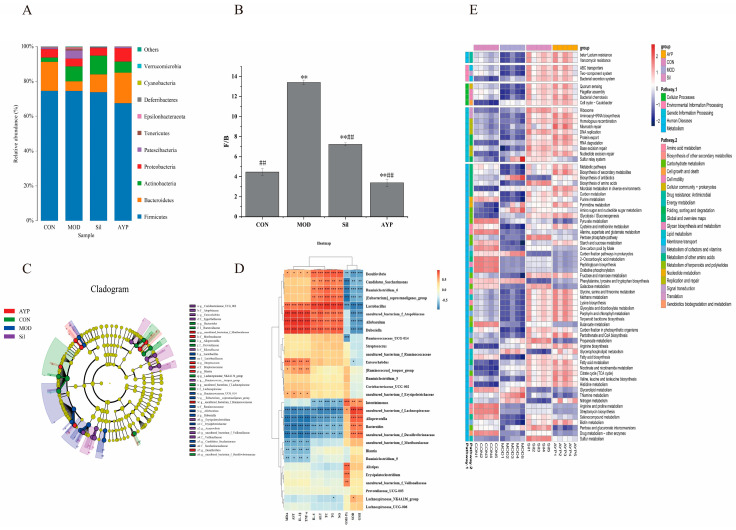
Effects of AYP on gut bacterial composition. Relative abundance of gut bacteria at the phylum level (**A**), ratio of F/B, ** *p* < 0.01 vs. CON; ## *p* < 0.01 vs. MOD. Duncan’s test was performed to determine the differences (**B**), LEfse statistical difference analysis cladogram (**C**). Genus relative abundance of mice at the level of cytokines, antioxidants and liver function parameters correlation analysis (**D**). The heatmap of functional prediction of altered gut bacteria by PICRUSt analysis based on KEGG (**E**). (* 0.01 < *p* < 0.05, ** 0.001 < *p* < 0.01, *** *p* < 0.001).

To determine the particular taxa associated with AYP, relative abundance at the phylum level was evaluated. F/B was the abundance ratio of *Firmicutes* and *Bacteroidetes*, which can indicate the overall microbial composition of the intestinal tract [51]. There was a highly significant difference the F/B values between the MOD group and the CON group. This phenomenon was consistent with previous studies in which ethanol feeding to mice significantly reduced the abundance of *Bacteroidetes* [52], while no significant changes were observed in the abundance of *Firmicutes* (Figure 6A), indicating that the intestinal ecosystem was disturbed. When treated with AYP by gavage, there was a highly significant difference in F/B values between the AYP group and the MOD group (*p* < 0.01), and the therapeutic effect of the AYP group was superior to the Sil group (Figure 6B).

LEfSe analysis is a species analysis technique used to identify enriched bacteria between groups and is primarily used to identify species with significant differences in abundance [53]. As shown in Figure 6C, *Lachnospiraceae* (*Lachnospiraceae_NK4A136_group* and *uncultured_bacterium_f_Lachnospiraceae*), *Bacteroidaceae* (*Bacteroides*) and *Prevotellaceae* (*Alloprevotella*), etc., were the characteristic bacteria in the CON group. *Veillonellaceae* (*uncultured_bacterium_f_Veillonellaceae*) was the characteristic bacteria in the Sil group. *Muribaculaceae* (*uncultured_bacterium_f_Muribaculaceae*) and *Streptococcaceae* (*Streptococcus*) were the specialized microorganisms in the AYP group. *Lactobacillaceae* (*Lactobacillus*), *Ruminococcaceae* (*Ruminococcaceae_UCG_014*), *Erysipelotrichaceae* (*Allobaculum*) *Saccharimonadaceae* (*Candidatus_Saccharimonas*) and *Dubosiella*, etc., were the specialized microorganisms in the MOD group. 

Meanwhile, the relationship between biochemical indicators and intestinal bacteria was evaluated using Spearman correlation analysis [54]. As shown in Figure 6D, nine genera were positively correlated with indices of liver function and cytokines, while seven genera were positively correlated with indices of antioxidants. The correlation heatmap showed that *Lactobacillus*, *Allobaculum*, *Dubosiella*, *Ruminococcaceae_UCG-014* and *Candidatus_Saccharimonas* were positively associated with the content of TC, TG, AST, ALT, NO and MDA in the liver and serum cytokines, whereas they were negatively related with the liver GSH, GSH-Px and SOD. The opposite results were reflected in *Bacteroides*, *uncultured_bacterium_f_Lachnospiraceae*, *Alloprevotella*, *Lachnospiraceae_NK4A136_group* and *uncultured_bacterium_f_Muribaculaceae*. 

Specific microbial signatures have the capability to differentiate distinct complications of alcohol consumption in alcoholic patients [55]. *Lachnospiraceae* and *Muribaculaceae* produce butyrate, which are powerful indicators of a healthy intestinal, produced butyrate [56,57,58]. A related report revealed that the incidence of ALD was significantly correlated with the abundance of *Lachnospiraceae* and *Muribaculaceae* [59]. *Uncultured_bacteria_f_Lachnospiraceae* and *Lachnospiraceae_NK4A136_group* were reported to have the potential ability to enhance the host antioxidant capacity [60], which coincides with the positive correlation between antioxidant indices observed in this investigation. Additionally, *uncultured_bacterium_f_Muribaculaceae* could inhibit the activation of CD8^+^ T cells to resist immune stimulation and correlate negatively with inflammation [61], which was enriched in the administration group. *Veillonellaceae* convert lactic acid into propionic acid and have anti-inflammatory properties [62]. *Alloprevotella* is able to stimulate SCFAs production and is inversely correlated with liver indicators and inflammatory factors [63]. Families of *Ruminococcaeae* are enriched with ardent drinkers. This microbiota signature indicates whether frequent consumers develop alcohol-related hepatitis [64]. Alcohol exposure increased *Ruminococcaceae_UCG-014* abundance in mice [56]. *Allobaculum* was multiply enriched in non-alcoholic fatty liver [57]. *Candidatus_Saccharimonas* was enriched in a type 2 diabetes mouse model and positively correlated with liver indices in agreement with our experiment [58]. *Dubosiella* was enriched in autoimmune hepatitis and positively correlated with liver indicators and inflammatory factors [65], which is in agreement with the present study. *Lactobacillaceae* is usually present in the intestines as a probiotic [66]. Interestingly, alcohol feeding resulted in an increase in the relative abundance of *Lactobacillus* [67]. A previous shotgun metagenomic analysis of ALD demonstrated that the increase in *Lactobacillus* was primarily attributable to oral species (such as *Lactobacillus salivarius*) and did not include *Lactobacillus rhamnosus* as well [68]. There are two possible mechanisms for the higher abundance of *Lactobacillus* in ALD, one being an alcohol-induced disturbance in bile acid metabolism [69,70], and the other attributed to its metabolic capacity, for example, the ability to metabolize ethanol [71]. In conclusion, we hypothesized that AYP exerted hepatoprotective effects against ALD by accelerating the abundance of helpful bacteria (*Lachnospiraceae* and *Muribaculaceae*) and diminishing the abundance of detrimental bacteria like *Lactobacillus*.

In addition, KEGG family was displayed in the PICRUSt software [72], which was displayed in the heatmap along with the significance and abundance of the leading 70 metabolic pathways as determined by Duncan’s test (*p* < 0.05). As shown in Figure 6E, alcohol upregulated nine metabolic pathways that were all downregulated by AYP, including amino sugar and nucleotide sugar metabolism, phenylalanine, tyrosine and tryptophan biosynthesis, propanoate metabolism, glycerophospholipid metabolism, thiamine metabolism, etc. Additionally, AYP reversed 39 metabolic pathways that alcohol downregulated, including purine metabolism, fatty acid biosynthesis, cysteine and methionine metabolism, starch and sucrose metabolism, glycerolipid metabolism, etc. Phenylalanine, tyrosine and tryptophan biosynthesis, glycerophospholipid metabolism and fatty acid biosynthesis had captured our attention.

The biosynthesis of phenylalanine, tyrosine and tryptophan was increased by alcohol [73,74]. Inflammatory factors such as TNF-α and IL-6 had a positive correlation with phenylalanine levels, which were reported to be produced by *Lactobacillus* [75]. Strongly associated with hepatic steatosis were microbial metabolites of aromatic amino acids (e.g., phenylalanine, tyrosine, tryptophan), specifically phenylacetic acid [76]. As a precursor to phenylacetic acid, elevated phenylalanine may be metabolized to phenylacetic acid and further contribute to liver disease [77]. The variation in amino acid levels may be attributable to increased protein degradation and oxidative stress [78]. Thus, AYP increased antioxidant activity (GSH-PX, GSH, SOD) and reduced inflammatory factors (IL-6, TNF-α) (Figure 5B,C), which may be related to the phenylalanine, tyrosine and tryptophan biosynthesis, especially the production of phenylalanine and metabolites by *Lactobacillus*.

Glycerophospholipid metabolism is regarded as metabolic pathway associated with liver injury phenotypes [79]. As the primary constituents of cell membranes, glycerophospholipids are crucial for cellular functions (such as molecular transport, protein function and signal transduction) associated with inflammation, metabolic syndrome and fibrosis [80]. The upregulated fecal levels of glycerophospholipids and their metabolites indicate that the cell membrane may be damaged. The damaged colon tissue may have contributed to the increased fecal glycerophospholipid levels [76]. SCFAs are catabolic products of fatty acids with various disease-preventive effects, whereas fatty acids are produced by intestinal bacteria and the downregulation of fatty acid biosynthesis may indicate disruption of intestinal bacteria [81]. We speculated that AYP may reverse alcohol-induced metabolic abnormalities by enriching the intestinal bacteria to produce SCFAs and other metabolites.

#### 2.5.2. The Effect of AYP on the Intestinal Fungi of Mice

Intestinal fungi also serve a crucial role in the pathogenesis of ALD [82]. To investigate the effect of AYP on intestinal fungi, ITS-1 rDNA sequencing was performed on mouse feces from each cohort. 

**Figure 7:**
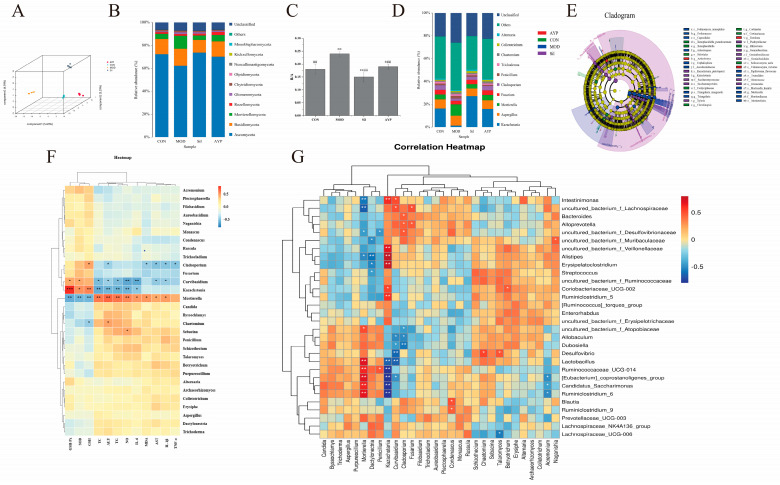
Effect of AYP on the intestinal fungi. Intestinal fungi based on OTU abundance PLS-DA (**A**), Relative abundance of intestinal fungi at the phylum level (**B**), ratio of B/A, * *p* < 0.05, ** *p* < 0.01 vs. CON; ## *p* < 0.01 vs. MOD (**C**). Duncan’s test was performed to determine the differences. Relative abundance of intestinal fungi at the genus level (**D**), LFfse statistical difference analysis cladogram (**E**). Genus relative abundance of mice at the level of cytokines, antioxidants and liver function parameters correlation analysis (**F**). Correlation analysis of the intestinal bacteria and fungi (**G**) (* 0.01 < *p* < 0.05, ** 0.001 < *p* < 0.01, *** *p* < 0.001).

Indicators of alpha diversity are shown in Table 2. The α index of the MOD group showed an increasing trend, consisting of reports that chronic ethanol intake increased fungal abundance and diversity [83], which indicate that alcohol changed the abundance of intestinal fungi while AYP reversed this trend. According to PLS-DA data (Figure 7A), there were no differences in clustering within groups, but there were differences between groups. The intestinal fungi composition of the AYP group was very different from that of the MOD group and similar to that of the CON group.

Investigation of the intestinal fungi in mice with ALD showed that the intestine was composed of three main fungi, including *Ascomycota*, *Basidiomycota* and *Mortierellomycota* (Figure 7B). The ratio of *Basidiomycota*/*Ascomycota* (B/A) is usually defined as an indicator of fungal dysbiosis [84]. The B/A of the MOD group was considerably different from that of the CON group, indicating that the intestinal fungi were destroyed by alcohol and that this ratio was restored after AYP and Sil administration (Figure 7C). Moreover, the abundance of *Mortierellomycota* was upregulated by alcohol and reversed by AYP. Juvenile ruminants are prone to diarrhea and *Mortierellomycota*, a fungus specific to juvenile yaks and positively associated with diarrhea in comparison to adult yaks [85], which was decreased in abundance after AYP treatment. 

In addition, the genus levels of *Kazachstania* and *Mortierella* also deserved our attention, with *Kazachstania* abundance decreasing significantly in the MOD group while *Mortierella* abundance increased significantly in the MOD group, while Sil and AYP reversed this trend (Figure 7D). Moreover, LEfSe analysis results showed that *Mortierellaceae* (*Mortierella*), *Saccharomycetaceae* (*Kazachstania*), *Arthrobotrys* and *Entoloma* were significantly enriched in the MOD group, the Sil group and the AYP group, respectively (Figure 7E). Furthermore, correlations between the relative abundance and cytokines, antioxidant and liver function parameters in mice of genus levels are shown in Figure 7F. *Kazachstania*, *Fusarium* and *Cladosporium* were negatively correlated with liver indicators, as well as cytokines, and positively correlated with antioxidant indicators, while *Mortierella*, *Candida* and *Chaetomium* showed the opposite trend. The downregulation of *Kazachstania* abundance in alcohol use disorder has been reported [86]. However, little research has been done on the function of *Kazachstania*, which is crucial for developing a healthy porcine microbiome and supporting the growth of SCFA-producing bacteria [87,88]. *Fusarium* and *Cladosporium* were altered due to alcohol intake in alcoholic liver disease and have anti-inflammatory and immunoprotective effects [83,89]. A high abundance of *Mortierella* was identified in calves with diarrhea [85]. However, the relative proportions of *Candida albicans* increased in patients with alcoholic hepatitis, which was positively correlated with AST levels and were rod cytotoxic, increasing mortality in mice [90]. Thus, AYP may be able to counteract alcohol-induced liver damage by adjusting the B/A ratio, adjusting the abundance of beneficial fungi, like *Kazachstania*, as well as harmful fungi, such as *Mortierella* and *Candida*. 

Fungi and bacteria interact intimately in the stomach, altering health and disease [82]. Bacteria and fungi are the most studied gastrointestinal microbiota in ALD, but their effects, mechanisms and cross-border interactions are still unknown. For instance, rodent models of ALD with AYP lack research on the interactions between intestinal bacteria and fungi. Consequently, we conducted a fungal–bacterial correlation investigation (Figure 7G) to ascertain how fungi and bacteria impacted AYP for ALD. At the genus level, the trans-kingdom association between intestinal bacteria and fungi with the top 30 abundance shown here was statistically significant (*p* < 0.05).

Among them, we focused on studying the correlation between bacteria and fungi which were regulated by AYP and related to physiological and biochemical indicators. Beneficial bacteria (*uncultured_bacterium_f_Muribaculaceae*, *uncultured_bacterium_Veillonellaceae*, *uncultured_bacterium_f_Lachnospiraceae* and *Alloprevptella*) enriched by AYP were negatively correlated with harmful fungi (*Mortierella*, *Byssochlamys* and *Dactylonectria*). Moreover, harmful bacteria (*Allobaculum*, *Lactobacillus* and *Ruminococcaceae_UCG-014*) were positively correlated with harmful fungi (*Candida*, *Moritierella*, *Penicillium* and *Aspergillus*) but negatively correlated with *Kazachstania*, *Fusarium*, *Curvibasidum* and *Cladosporium*. *Kazachstania* was positively related to the beneficial intestinal bacteria community (*uncultured_bacterium_f_Muribaculaceae*, *uncultured_bacterium_Veillonellaceae*, *uncultured_bacterium_f_Lachnospiraceae*, *Alistipes* and *Erysipelatoclostridium*), suggesting that there might be a co-dependence for both kingdoms. These negatively correlated intestinal microbiota may have a competitive or inhibitory role in the mitigation mechanism of AYP on alcoholic liver injury. 

*Kazachstania slooffiae* is a pig-specific species of intestinal fungus that may play a significant function in host health [91] and can produce some bioactive substances, such as peptides, formic acid and dehydroascorbic acid [92]. *Candida albicans* (*C. albicans*) is a commensal fungus in the human intestinal tract, and its relative proportion was elevated in patients with AUD and alcoholic hepatitis patients [93], where albicans interacted with bacteria through mechanisms involving the formation and alteration of biofilms and competition with *Lactobacillus rhamnosus* GG [94,95]. The abundance of *Moritierella* was negatively correlated with *Alloprevotella*, *uncultured_bacterium_Veillonellaceae*, *uncultured_bacterium_f_ Lachnospiraceae* and *Ruminococcaceae_UCG-014*. The abundance of beneficial bacteria such as *Alloprevotella*, *Veillonella*, *uncultured bacteria_f_Lachnospiraceae* and *Ruminococcus_UCG-014* were significantly reduced in the intestine of Bauer’s pigeons with diarrhea [96]. These results suggest extensive co-variation associations between intestinal bacteria and fungi, including those that might be involved in the process of AYP alleviating alcohol damage. However, to confirm whether intestinal bacteria and fungi are new targets for the AYP treatment of ALD and to elucidate their potential cause–effect relationships and bacteria and fungi interactions, more experiments, such as FMT, co-housing, and germ-free mice, will be conducted in our follow-up study.

## 3. Materials and Methods

### 3.1. Materials and Reagents

*A. cornea* var. Li. was provided and identified by Prof. Qi Wang (Engineering Research Center of Edible and Medicinal Fungi, Ministry of Education, Jilin Agricultural University). Silymarin was acquired from a neighborhood pharmacy (Tasly Sants, Tianjin, China.). AST (aspartate aminotransferase, C010-2-1), ALT (alanine aminotransferase, C009-2-1), TG (triglyceride, A110-1-1), TC (total cholesterol, A111-1-1), NO (nitric oxide, A013-2-1), MDA (malondialdehyde, A003-1-2), SOD (superoxide dismutase, A001-3-2), GSH-Px (glutathione peroxidase, A005-1-2), GSH (glutathione, A006-2-1) and TP (total protein, A045-4-2) kits were purchased from Nanjing Jiancheng Bioengineering Institute (Nanjing, China). IL-6 (interleukin-6, MU30044), IL-1β (interleukin-1β, MU30369) and TNF-α (tumor necrosis factor-α, MU30030) ELISA kits were obtained from the Bioswamp Company (Wuhan, China). The rest of the pharmaceuticals and reagents were of analytical grade.

### 3.2. Extraction and Purification

The crude polysaccharide was obtained referencing the method of previous experimental report with slight modifications [4], and the crude polysaccharide was purified to obtain the AYP. *A. cornea* var. Li particles were extracted for three hours with a 0.4% pectinase solution (solid–liquid ratio of 1:80 g/mL, pH 5, extraction temperature of 50 °C) and then immersed for one hour in 85 °C water. The concentration of the supernatant was then precipitated with ethanol. After deproteinization using the Sevag method and dialysis [97], unprocessed polysaccharides were obtained. The crude polysaccharides were purified with DEAE-52 and Sephacryl S-400, then freeze-dried to produce an amorphous polysaccharide.

### 3.3. Structural and Polysicochemical Analysis of AYP 

Total polysaccharide content and reducing sugar content were separately determined by the phenol-sulfuric acid method [98] and 3, 5-dinitrobenzene sulfonic acid (DNS) method [99]. Total protein content was determined using the Coomassie bright blue G250 assay [100]. Meanwhile, the chemical composition of AYP was determined by measuring the absorbance by the microplate reader (SPARK 10M, TECAN, Zurich, Switzerland). The molecular weight of AYP was determined using high-performance gel permeation chromatography (HPGPC-ELSD). Monosaccharide composition was analyzed by ion exchange chromatography [101]. AYP and KBr were uniformly combined and pressed into transparent particles for Fourier-transform infrared spectrometer (FT-IR) analysis [102].

### 3.4. Animal Experiment

#### 3.4.1. Experimental Animals and Procedures

**Figure 8:**
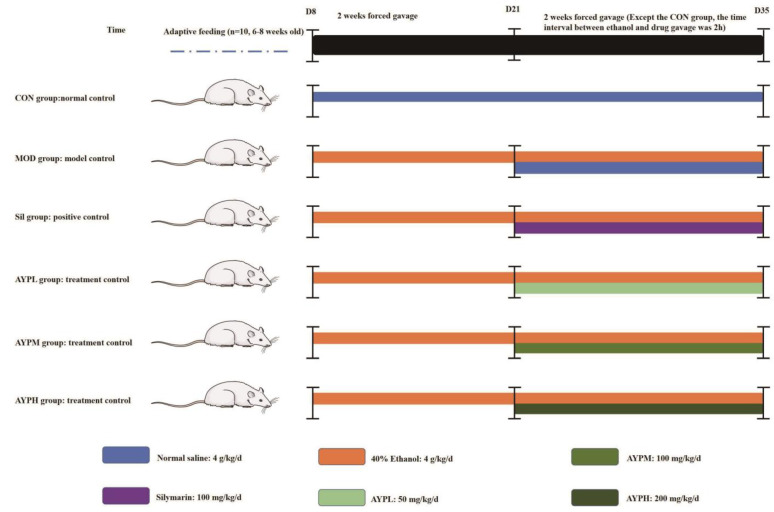
Grouping diagram of the animal experiments.

The animal experiments were supervised by the Ethics Committee for Laboratory Animals at Jilin Agricultural University and conducted in accordance with the China Animal Welfare Legislation (ethical approval code: 20230317001). In total, 60 SPF-grade Kunming male mice (18–22 g) were obtained from Liaoning Changsheng Biotechnology Co., Ltd. (License number: SCXK (Liao) 2015-0001). Mice were maintained with a specific diet and unfettered access to water at 22 ± 2 °C, humidity levels of 50% and a 12 h light and 12 h dark cycle. The alcohol liver model and polysaccharide administration method were utilized to conduct the animal experimentation [5] (Figure 8). In brief, after acclimatization to standard laboratory conditions for one week, all the mice were divided into six groups, with ten mice in each group as follows: the CON group—mice treated with saline as a negative control, the MOD group—ALD of mice as a model control, the Sil group—ALD of mice treated with 100 mg/kg/d Silymarin as a positive control, the AYPL group—ALD of mice treated with 50 mg/kg/d AYP, the AYPM group—ALD of mice treated with 100 mg/kg/d AYP, the AYPH group—ALD of mice treated with 200 mg/kg/d AYP. In the first two weeks, all the mice except for those in the CON group were forced to gavage with 40% ethanol. In the following two weeks, 40% ethanol was gavaged in all groups except the CON group, and after two hours, each group was gavaged with the appropriate therapeutic drug.

After the final gavage, rodents were fasted alone for 12 h before being euthanized for the collection of serum, liver and cecum. The liver and the contents of the cecum were promptly gathered and frozen at −80 °C. The study protocol on experimental animals in this research has been registered at https://preclinicaltrials.eu (27 November 2023) under the registration number PCTE0000437.

#### 3.4.2. Measurements of Liver Damage

The volume ratio of liver tissue to 0.9% sodium chloride solution was 1:9, with sufficient grinding at a low temperature and high speed. According to the kit’s instructions, the ALT, AST, TG and TC levels in the supernatant were measured after centrifugation at low temperatures [103]. Fixed in 10% paraformaldehyde, the mouse liver samples were processed to obtain 5 μm paraffin-embedded sections. The sections were stained with hematoxylin and eosin (H&E).

#### 3.4.3. Measurements of Liver Oxidative Stress, and Serum Cytokines

The volume ratio of liver tissue to 0.9% sodium chloride solution was 1:9, and the supernatant was taken by centrifugation after sufficient grinding at a low temperature and high speed. According to the kit’s instructions, the levels of NO, MDA and the activities of SOD, GSH and GSH-Px in the supernatant were measured after determination of protein content by TP kit. Serum levels of TNF-α, IL-6 and IL-1β were measured through an ELISA kit based on the instructions supplied by the manufacturer [104].

#### 3.4.4. Analysis of the Intestinal Bacteria and Fungi

Genomic DNA were obtained from the contents of the cecum with the PowerSoil DNA Isolation Kit (MO BIO Laboratories, Carlsbad, CA, USA). Each sample had three replicates that were measured. Biomarker Technologies selected the Illumina Novaseq technology to amplify and sequence the V3-V4 regions of 16S rDNA and ITS-1 (Beijing, China) [27,60].

### 3.5. Statistical Analysis

The results were analyzed using Duncan’s test in the one-way ANOVA of SPSS software and expressed as the mean (standard error of the mean (SEM)) (26.0). *p* < 0.01 was considered extremely statistically significant, and *p* < 0.05 was considered statistically significant. For subsequent data processing, GraphPad Prism 9 and Origin 2021 were utilized.

## 4. Conclusions

In this study, we obtained a new type of polysaccharide (AYP) with a clear structure and a protective effect against ALD. Moreover, we explored the effects of AYP on ALD from the analysis of intestinal bacteria, fungi and their correlation for the first time, which lays the experimental foundation for the prevention of alcoholic-related diseases by natural polysaccharides and opens up a new direction. Indeed, we will use electron microscopic scanning, X-ray diffraction method, nuclear magnetic resonance, atomic force spectroscopy and molecular dynamics simulation technology to identify the final structure of purified AYP in the future. At the same time, we will also use the proteome and metabolomics to elucidate the molecular mechanism of AYP protection from ALD and further elucidate its structure–activity relationship with AYP in future research.

## Figures and Tables

**Table 1:** α-diversity indexes of intestinal bacteria.

	ACE	Chao1	Simpson	Shannon
CON	521.429 ± 14.231	533.166 ± 9.466	0.979 ± 0.004 ^##^	6.645 ± 0.178 ^##^
MOD	533.133 ± 13.329	538.946 ± 10.888	0.961 ± 0.003 **	6.326 ± 0.114 **
Sil	464.096 ± 10.631 **^##^	468.206 ± 13.210 **^##^	0.969 ± 0.000 **^##^	6.235 ± 0.01 **
AYP	481.839 ± 7.224 **^##^	484.193 ± 6.489 **^##^	0.981 ± 0.0027 ^##^	6.890 ± 0.056 *^##^

Data are expressed as mean ± SD (n = 5). * *p* < 0.05, ** *p* < 0.01 vs. MOD. ## *p* < 0.01 vs. CON.

**Table 2:** α-diversity indexes of intestinal fungi.

	ACE	Chao1	Simpson	Shannon
CON	595.656 ± 127.861	454.586 ± 112.503	0.967 ± 0.007 *	6.680 ± 0.117 **
MOD	647.149 ± 294.838	506.445 ± 130.516	0.986 ± 0.0106	7.169 ± 0.228
Sil	715.839 ± 96.040	389.859 ± 30.940	0.923 ± 0.011 **	6.109 ± 0.133 **
AYP	604.215 ± 135.579	405.881 ± 54.869	0.967 ± 0.014 *	6.730 ± 0.258 *

Data are expressed as mean ± SD (n = 5). * *p* < 0.05, ** *p* < 0.01 vs. MOD.

## Data Availability

The data that support the findings of this study are available from the corresponding authors upon reasonable request.

## Supplementary Materials

The following supporting information can be downloaded at: https://www.mdpi.com/article/10.3390/molecules28248003/s1. Figure S1. α diversity of intestinal bacteria. Rarefaction Curve (A), Shannon index curve (B), Rank abundance curve (C). β diversity analysis of intestinal bacteria. Partial Least Squares Discriminant Analysis (D), Principal Component Analysis (E).
