# Supplementary material for: The Protective Effect of Auricularia cornea var. Li. Polysaccharide on Alcoholic Liver Disease and Its Effect on Intestinal Microbiota
Source: Molecules. 2023 Dec 8;28(24):8003. doi: 10.3390/molecules28248003 (PMC10745760; doi:10.3390/molecules28248003)
Supplement: Supplementary file 1 [file molecules-28-08003-s001.zip › molecules-2738946-supplementary.pdf]

A

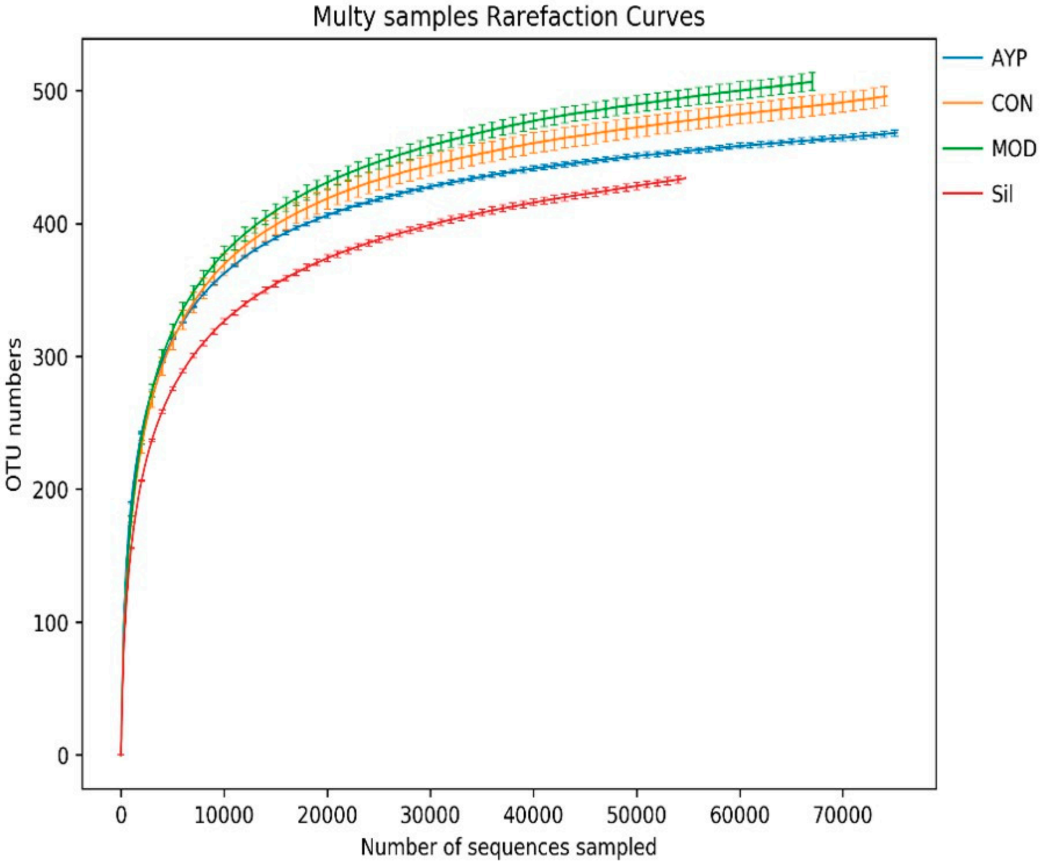

Multy samples Rarefaction Curves
AYP
CON
MOD
Sil
OTU numbers
Number of sequences sampled
0
100
200
300
400
500
0
10000
20000
30000
40000
50000
60000
70000

B

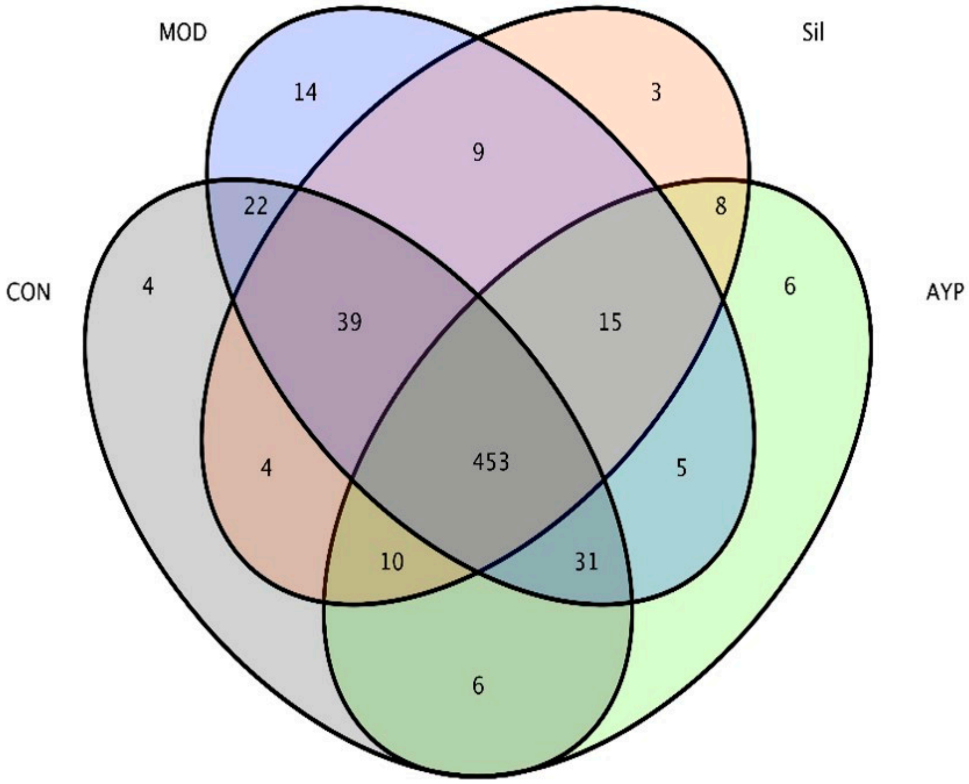

C

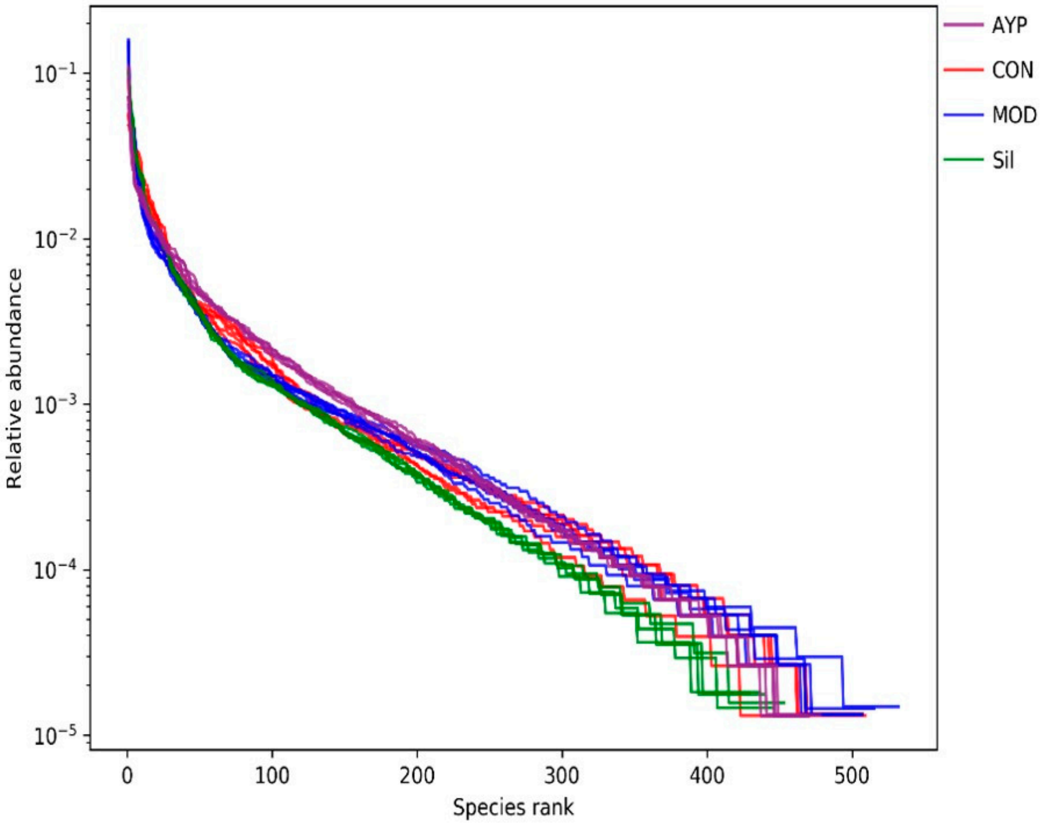

AYP
CON
MOD
Sil
Relative abundance
10^{-1}
10^{-2}
10^{-3}
10^{-4}
10^{-5}
0
100
200
300
400
500
Species rank

D

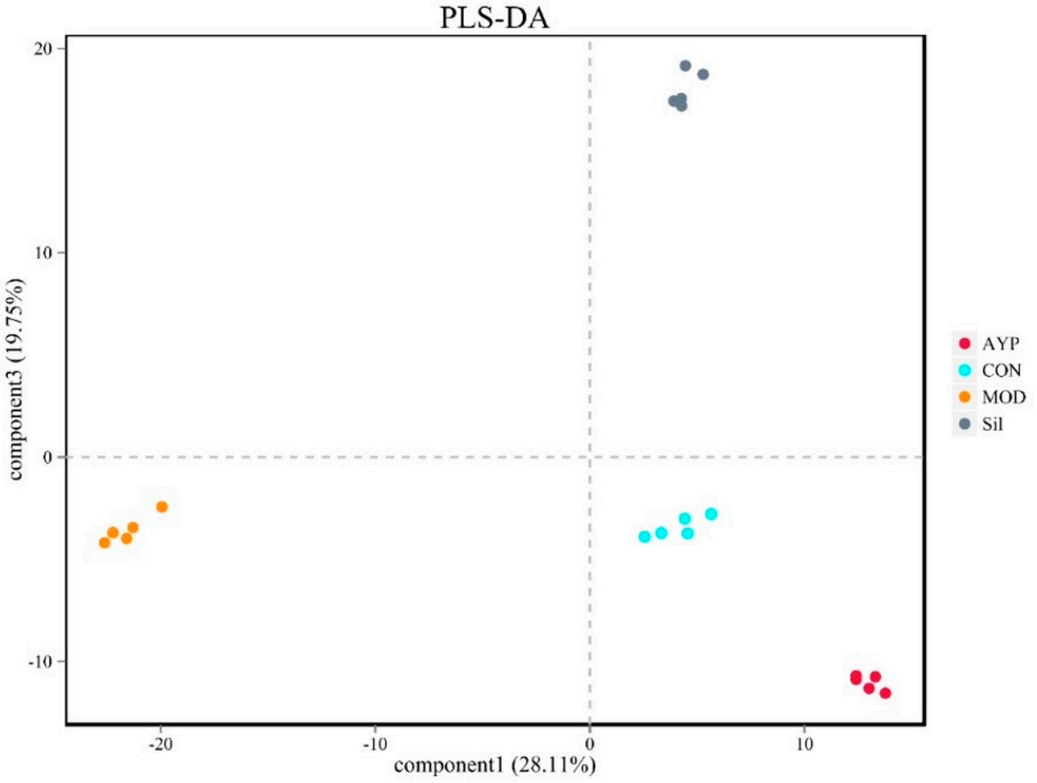
PLS-DA
20
10
0
-10
-20
-10
0
10
component1 (28.11%)
component3 (19.75%)
AYP
CON
MOD
Sil

E

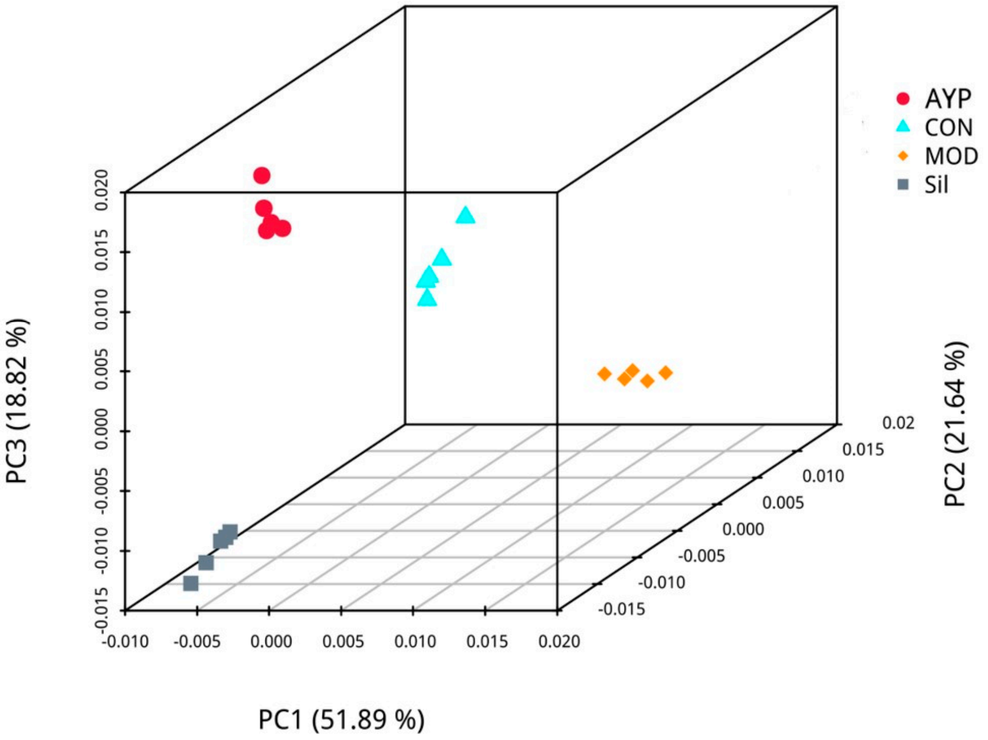

Figure S1.

Figure S1. α diversity of intestinal bacteria. Rarefaction Curve(**A**), Shannon index curve(**B**), Rank abundance curve(**C**). β diversity analysis of intestinal bacteria. Partial Least Squares Discriminant Analysis(**D**), Principal Component Analysis(**E**).
